# Predictors for length of hospital stay in patients with community-acquired Pneumonia: Results from a Swiss Multicenter study

**DOI:** 10.1186/1471-2466-12-21

**Published:** 2012-05-20

**Authors:** Isabelle Suter-Widmer, Mirjam Christ-Crain, Werner Zimmerli, Werner Albrich, Beat Mueller, Philipp Schuetz

**Affiliations:** 1 Department of Internal Medicine, Division of Endocrinology, Diabetes and Metabolism, University Hospital Basel, Basel, Switzerland; 2 Medical University Clinic, Kantonsspital Liestal, Liestal, Switzerland; 3 Medical University Clinic, Kantonsspital Aarau, Aarau, Switzerland; 4 Harvard School of Public Health, Boston, USA

## Abstract

**Background:**

Length of hospital stay (LOS) in patients with community-acquired pneumonia (CAP) is variable and directly related to medical costs. Accurate estimation of LOS on admission and during follow-up may result in earlier and more efficient discharge strategies.

**Methods:**

This is a prospective multicenter study including patients in emergency departments of 6 tertiary care hospitals in Switzerland between October 2006 and March 2008. Medical history, clinical data at presentation and health care insurance class were collected. We calculated univariate and multivariate cox regression models to assess the association of different characteristics with LOS. In a split sample analysis, we created two LOS prediction rules, first including only admission data, and second including also additional inpatient information.

**Results:**

The mean LOS in the 875 included CAP patients was 9.8 days (95%CI 9.3-10.4). Older age, respiratory rate >20 pm, nursing home residence, chronic pulmonary disease, diabetes, multilobar CAP and the pneumonia severity index class were independently associated with longer LOS in the admission prediction model. When also considering follow-up information, low albumin levels, ICU transfer and development of CAP-associated complications were additional independent risk factors for prolonged LOS. Both weighted clinical prediction rules based on these factors showed a high separation of patients in Kaplan Meier Curves (*p logrank <0.001 and <0.001*) and a good calibration when comparing predicted and observed results.

**Conclusions:**

Within this study we identified different baseline and follow-up characteristics to be strong and independent predictors for LOS. If validated in future studies, these factors may help to optimize discharge strategies and thus shorten LOS in CAP patients.

## Background

Community-acquired pneumonia (CAP) is a major cause for hospitalization and has a substantial impact on health care costs [1]. Therefore, economic and rational management of CAP should include discharge of patients as early as possible without exposing them to the risk for worsening or recurrent infection. Current guidelines recommend discharging patients as soon as they are clinically stable, have no other active medical problems, and have a safe environment for continued care [2]. Still, length of hospital stay (LOS) is variable and arbitrary in different settings. For example, in Switzerland LOS in patients with CAP was only reduced by 1.2 days over the last 10 years [3]. In an ageing patient population, social, functional and nursing-related factors may become increasingly important and early discharge planning including organization of help and/or nursing assistance at home becomes more important for shortening LOS [4]. In this context, early information of patients and their families about time management and expected duration of LOS is of particular importance and may help to optimize early discharge strategies.

There are a number of well validated clinical scores which predict 30-day mortality and have shown to improve initial-site-of-care decisions [5-7]. However, there is no similar instrument for prediction of LOS. Previously, different parameters which are associated with LOS have been reported [8-11]. Some predictors directly relate to the acute disease or underlying comorbidities, while other factors include the social situation of the patient. Yet, a reliable clinical prediction rule which helps physicians estimating LOS based on these and other factors is missing.

The aim of the present study was to propose a LOS prediction rule in a well characterized cohort of CAP patients from six different Swiss hospitals, one only focusing on admission parameters and one also considering follow up information. Furthermore, we investigated whether type of medical insurance was associated with LOS. One could assume that privately insured patients have shorter LOS because they may have priority for diagnostic studies, are more easily being transferred to post acute care centers and are treated by more experienced physicians. On the other hand, financial incentives could lead to higher LOS in patients, particularly in a pay-for-service health care system such as in Switzerland.

## Methods

### Study design and setting

Herein, we used the clinical data from all patients with CAP enrolled in the multicenter ProHOSP study [12]. The design of this study has been reported in detail elsewhere [12]. In brief, from October 2006 to March 2008, a total of 1359 consecutive patients with presumed lower respiratory tract infection (LRTI) from six different hospitals located in the northern part of Switzerland were included. In total, 925 patients had the definite diagnosis of CAP. Patients were randomly assigned to an intervention group, where guidance of antibiotic therapy was based on procalcitonin cut-off ranges, and to a standard group in which guidance of antibiotic therapy was based on enforced guideline recommendations without knowledge of procalcitonin. The baseline characteristics for both groups were similar and both were treated according to current guidelines.

### Selection of participants

Patients had to be over 18 years old with a definite diagnosis of CAP to be eligible for this analysis. Inclusion criteria included the presence of at least 1 respiratory symptom (cough, sputum production, dyspnea, tachypnea, pleuritic pain), plus at least 1 finding during auscultation (rales, crepitation), or 1 sign of infection (core Body temperature >38.0°C, shivering, or Leukocyte count >10000/μL or < 4000/μL) independent of antibiotic pretreatment. CAP was defined as a new infiltrate on chest radiograph. Exclusion criteria included patients with active intravenous drug use, severe immunosuppression other than corticosteroid use, life-threatening medical comorbidities leading to possible imminent death, patients with hospital acquired pneumonia and patients with chronic infection necessitating antibiotic treatment. We only included patients who survived their hospital stay for this analysis, as early death would induce a bias for LOS prediction. Patients were examined on admission to the emergency department by a resident supervised by a board-certified specialist in internal medicine. The standardized baseline assessment included medical history, clinical examination, lab tests and chest X-ray. For all patients with CAP, the PSI and the CURB65 was calculated on admission as described elsewhere [6,7]. The study protocol was approved by all local ethical committees, and written informed consent was obtained from all participants.

### Study endpoints and selection of covariates

The primary endpoint of this analysis is LOS defined as the time from hospital admission to hospital discharge. We recorded baseline data including demographic characteristics (age, gender), comorbid diseases, and living situation (at home, nursing home residents or living with continuous nursing support at home), insurance status, clinical presentation on admission and initial results from blood analysis. All patients were followed until hospital discharge and we further assessed adverse events during the hospital stay including need for ICU admission and development of CAP specific complications (empyema).

### Statistical analysis

Variables are presented as medians and interquartile range and a two group comparison was made with Wilcoxon-MWU tests. In a first step, we assessed the association of different baseline characteristics with LOS in univariate Cox regression models with time to hospital discharge of patients as the endpoint of interest. For the time to event analysis, patients were censored at the time of hospital discharge. Further, we calculated multivariate Cox regression analysis adjusted for all covariates to assess which parameters had an independent association with longer LOS. Of note, hazard ratios (HRs) lower than 1 correspond to an association of the factor with longer LOS, while high HRs correspond to earlier discharge. The proportional hazards assumption of Cox regression models was evaluated by graphical display and analysis of the scaled Schoenfeld residuals as recommended [13]. In a 50:50 split sample analysis, we used all independent predictors from the multivariate model to calculate two weighted clinical decision rules assigning points to each predictor based on the magnitude of association (i.e. HR 0.8-0.9 = 1 point, 0.7-0.8 2 points, 0.6-0.5 = 3 points, <0.5 = 4 points); the prediction of based on the first 50 % of CAP patients was then used to predict LOS in the second half of patients to assess calibration. We developed two models: a first one considered baseline criteria only, and a second model also considered factors during follow up. To illustrate the association of this rule and LOS, we displayed data in Kaplan Meier survival curves stratified by the number of predictors.

In a sensitivity analysis we also investigated whether randomization of the initial study had an effect on this analysis. We found no association of the intervention on LOS (HR 1.05, 95%CI 0.92-1.20). Also, when including an interaction term into the regression model, we found no evidence of effect modification. Therefore treatment assignment was not further considered in this analysis.

All testing was two-tailed and P values less than 0.05 were considered to indicate statistical significance. All calculations were performed using STATA 9.2 (Stata Corp, College Station, Texas).

## Results

### Patient population

From a total of 925 patients, 875 (94.6 %) patients survived their hospital stay and were included in the further analysis. Baseline characteristics of the study cohort are presented in Table 1. The median age of the patients at the time of study enrolment was 72 years and 42 % were women. Patients had important comorbidities including chronic pulmonary disease in 29 %, congestive heart failure in 16 % and diabetes in 17 %. A total of 49 % of patients were classified in high risks PSI classes IV or V.

**Table 1:** Baseline characteristics and outcomes of surviving CAP patients (n = 875)

**Characteristics**	**Value** n = 875
**Demographic characteristics**	72 (57–82)
Age (years)	511 (58 %)
Sex (male) - no. (%)	
**Past Medical history**	291 (33 %)
History of chills	628 (72 %)
Former or current smoker	326 (37 %)
Regular alcohol consumption	
**Social situation at home**	580 (66 %)
Lives independently	295 (34 %)
Nursing home resident or living with continuous nursing help at home	
**Coexisting illnesses** - no. (%)	143 (16 %)
Chronic heart failure	258 (29 %)
Chronic pulmonary disease	152 (17 %)
Diabetes	107 (12 %)
Malignancy	
**Clinical findings**	64 (7 %)
Confusion - no. (%)	313 (36 %)
Respiratory rate > 20 breaths/minute	24 (3 %)
Systolic blood pressure <100 mmHg	312 (36 %)
Heart rate > 100 beats/minute	328 (37 %)
Body temperature > 38.5°C	
**PSI points**	92 (68–116)
**PSI class**- no. (%)	450 (51 %)
I, II, III	323 (37 %)
IV	102 (12 %)
V	

Data are presented as medians (IQR) or absolute numbers (%).

### Predictors for LOS in univariate models

A total of 5 % of patients were not hospitalized. The mean LOS of the overall cohort was 9.8 days (95%CI 9.3-10.4). We assessed LOS in Cox regression models where low HRs correspond to an association of the factor with longer LOS. Older age, respiratory rate >20 pm, confusion, residing in a nursing home, or need for regular nursing assistance at home, as well as different pre-existing comorbid conditions including chronic pulmonary disease, congestive heart failure and diabetes showed a significantly decreased chance to be discharged from the hospital in the univariate Cox regression analysis (Table 2). Furthermore, markers of high disease severity such as high procalcitonin levels >0.5ug/L, high CRP levels >150 mg/dL and PSI class were associated with longer time until hospital discharge. Similarly, positive blood cultures and multilobar CAP, as well as development of empyema or admission to the ICU during the hospital stay were also significantly associated with higher LOS.

**Table 2:** Univariate cox regression model for time to hospital discharge

**Parameters**	**HR (95%CI)**	***p***
**Demographics**		
Female Gender	0.95 (0.83-1.08)	*0.41*
**Age***(age <60 Reference group)*		
Age 60–70 years	0.63 (0.52-0.77)	***<0.001***
Age 70–80 years	0.52 (0.43-0.63)	***<0.001***
Age >80 years	0.46 (0.38-0.55)	***<0.001***
**Past medical history**		
Fever (Temp > 38.5°C)	0.82 (0.71-0.95)	***0.01***
Active smoker	0.85 (0.73-0.98)	***0.03***
Regular alcohol consumption	1.15 (1.01-1.31)	***0.05***
**Clinical presentation**		
BP systolic < 90 mmHg	0.67 (0.44-0.99)	***0.05***
BP diastolic < 60 mmHg	0.77 (0.54-10.7)	*0.22*
Pulse > 100 bpm	0.93 (0.81-1.07)	*0.32*
Temperature >38.5 or < 36°C	1.01 (0.88-1.15)	*0.93*
Respiratory rate > 20 bpm	0.72 (0.62-0.82)	***<0.001***
Confusion	0.76 (0.59-0.99)	***0.04***
**Situation at home**		
Nursing home or needing regular nursing assistance at home	0.70 (0.61-0.81)	***<0.001***
**Comorbid condition**		
Chronic pulmonary disease	0.75 (0.65-0.86)	***<0.001***
Chronic heart failure	0.68 (0.57-0.81)	***<0.001***
Diabetes	0.8 (0.67-0.95)	***<0.001***
Tumor	0.89 (0.73-1.09)	*0.22*
Chronic renal failure	0.73 (0.62-0.86)	***<0.001***
**Type of medical Insurance**		
Private insurance	1.1 (0.93-1.3)	*0.29*
**Initial blood analysis**		
Procalcitonin *(<0.1 ng/L Reference group)*		
Procalcitonin 0.1-0.25 *ng/L*	0.97 (0.77-1.21)	*0.76*
Procalcitonin 0.25-0.5 *ng/L*	0.78 (0.6-1.01)	*0.06*
Procalcitonin >0.5 *ng/L*	0.69 (0.56-0.85)	***<0.001***
**C-reactive protein***(<50* mg/L *Reference group)*		
C-reactive protein 50–100 mg/L	0.76 (0.6-0.96)	***0.02***
C-reactive protein 100–150 mg/L	1.03 (0.82-1.28)	*0.82*
C-reactive protein > 150 mg/L	0.78 (0.65-0.94)	***0.01***
Initial albumin level <30 g/L	0.80 (0.67-0.96)	***0.02***
Initial sodium <130 or >140 mmol/L	0.89 (0.76-1.04)	*0.17*
**Severity of CAP**		
Bacteremic CAP	0.74 (0.58-0.95)	***0.02***
Multilobar CAP	0.73 (0.56-0.93)	***0.01***
Development of empyema	0.41 (0.28-0.6)	***<0.001***
ICU transfer during hospitalisation	0.45 (35–0.59)	***<0.001***
**Pneumonia severity index**		
PSI class (per increase in PSI class)	0.71 (0.66-0.75)	***<0.001***

Of note, hazard ratios (HRs) lower than 1 correspond to an association of the factor with longer LOS, while high HRs correspond to earlier discharge.

### Predictors for LOS in multivariate models

**Figure 1:**
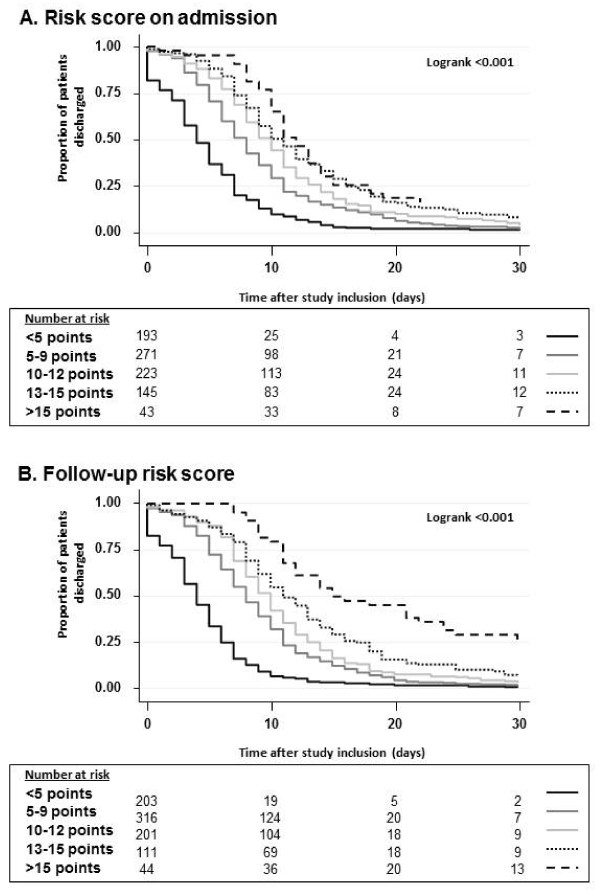
**Association of factors present on hospital admission and duration of hospital stay.** Points refer to a weighted-risk score based on age (3 points), high respiratory rate >20 pm (1 point), being a nursing home resident or need for regular outpatient nursing assistance at home (1 point), chronic pulmonary disease (1 point) and congestive heart failure (1 point) and multilobar CAP (1 point).

**Figure 2:**
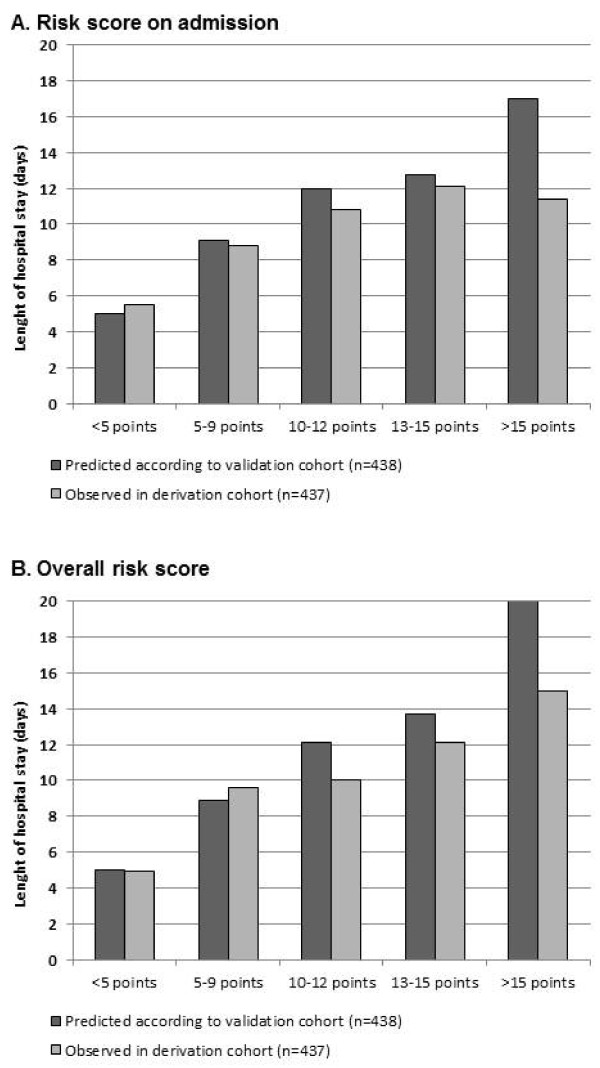
**Calibration of prediction rule on admission (A) and during follow up (B): predicted LOS from derivation cohort and observed LOS in validation cohort.** Points refer to a weighted-risk score based on age (3 points), high respiratory rate >20 pm (1 point), being a nursing home resident or need for regular outpatient nursing assistance at home (1 point), chronic pulmonary disease (3 point), ICU transfer during hospitalization and development of empyema (3 point).

Using a 50:50 split sample analysis approach, we calculated two multivariate prediction models: one for factors being present on hospital admission only, and one with all factors on admission and during hospital stay (Table 3). In the admission only model, older age, respiratory rate >20 pm, nursing home residence, chronic pulmonary disease, diabetes, multilobar CAP and the pneumonia severity index class were independently associated with longer LOS. We calculated a weighted prediction score assigning points based on the magnitude of association. This was also confirmed in Kaplan Meier curves for time to hospital discharge, where more points were associated with significantly longer time until hospital discharge (*p logrank <0.001*) (Figure 1). This prediction rule also showed a good calibration when used in the validation cohort (Figure 2).

**Table 3:** Multivariate models for prediction of Length of stay

**Parameters**	**Initial Prediciton model**			**Follow-up Prediciton model**		
	**HR (95%CI)**	***p***	**Points**	**HR (95%CI)**	***p***	**Points**
**Age***(age <60 Reference group)*						
Age 60–70 years	0.81 (0.59-1.11)	0.187	1	0.74 (0.54-1.01)	0.06	2
Age 70–80 years	0.65 (0.46-0.92)	0.014	3	0.59 (0.42-0.82)	0.002	4
Age >80 years	0.65 (0.46-0.92)	0.015	3	0.53 (0.37-0.76)	<0.001	4
**Past medical history**						
Fever (Temp > 38.5°C)	1.1 (0.89-1.36)	0.372		1.16 (0.93-1.44)	0.181	
Active smoker	1.04 (0.82-1.33)	0.745		0.98 (0.76-1.24)	0.839	
Regular alcohol consumption	1.22 (0.89-1.51)	0.261		1.36 (0.91-1.69)	0.34	
**Clinical presentation**						
BD systolic < 90 mmHg	0.78 (0.46-1.32)	0.348		1.29 (0.74-2.25)	0.362	
Respiratory rate > 20 pm	0.76 (0.61-0.95)	0.014	2	0.74 (0.6-0.92)	0.006	2
Confusion	0.99 (0.69-1.41)	0.939		1.03 (0.72-1.48)	0.855	
**Situation at home**						
Nursing home*	0.84 (0.67-1.06)	0.138	1	0.72 (0.57-0.92)	0.008	2
**Comorbid condition**						
Chronic pulmonary disease	0.70 (0.56-0.87)	<0.001	3	0.65 (0.52-0.81)	<0.001	3
Congestive heart failure	0.94 (0.71-1.24)	0.645		0.94 (0.71-1.24)	0.655	
Diabetes	0.77 (0.59-1.01)	0.062	2	0.68 (0.51-0.89)	0.006	3
renal failure	0.99 (0.76-1.29)	0.956		1.19 (0.9-1.56)	0.214	
**Initial blood analysis**						
						
Procalcitonin 0.1-0.25 *ng/L*	1.36 (0.97-1.89)	0.072		1.31 (0.94-1.84)	0.111	
Procalcitonin 0.25-0.5 *ng/L*	1.77 (0.88-2.64)	0.151		1.86 (0.85-2.79)	0.221	
Procalcitonin >0.5 *ng/L*	1.12 (0.8-1.58)	0.5		1.24 (0.88-1.76)	0.217	
						
C-reactive protein 50–100 mg/dl	0.89 (0.63-1.28)	0.537		0.97 (0.68-1.39)	0.882	
C-reactive protein 100–150 mg/dl	1.06 (0.76-1.46)	0.735		1.07 (0.77-1.48)	0.701	
C-reactive protein > 150 mg/dl	0.76 (0.57-1.01)	0.057		0.87 (0.65-1.15)	0.325	
**Nutrition marker**						
Initial albumin level <30 g/dl	0.81 (0.6-1.08)	0.153		0.77 (0.58-1.03)	0.082	2
**Severity of CAP**						
Multilobar CAP	0.68 (0.45-1.03)	0.066	3	0.92 (0.61-1.38)	0.684	
Pneumonia severity index (per increase in class)	0.79 (0.69-0.91)	<0.001	2	0.81 (0.7-0.93)	0.003	1
**Follow up history not available on admission**						
Positive blood cultures				0.89 (0.62-1.27)	0.509	
ICU transfer during hospitalisation				0.43 (0.29-0.66)	<0.001	5
Development of empyema				0.33 (0.19-0.57)	<0.001	6

*or needing regular nursing assistance at home.

An overall model including not only baseline factors, but also follow up information, namely blood culture results, need for ICU admission and development of complications (empyema) was also calculated. Both, need for ICU admission and development of complications (empyema), as well as initial albumin level were strong LOS predictors while multilobar CAP did not reach statistical significance in this second model. A weighted score based on these factors showed again a high discrimination of LOS and a good calibration when used in the validation cohort.

## Discussion

We identified several factors on admission and during follow-up, which were independently associated with longer LOS in patients with CAP. Integrated into a clinical prediction rule, these factors were able to predict LOS in a split sample analysis and showed high separation of LOS.

Previously different predictors for LOS have been reported [5,8-11]. Some predictors directly relate to the acute disease or underlying comorbidities, while other factors focus on the social situation of the patient. Predictors for LOS associated with the acute infection were abnormal blood results (low PaO2, low albumin, sodium imbalance), clinical signs of severity (low diastolic blood pressure, respiratory acidosis, high fever, confusion) or severity markers such as pleural effusion, multilobar lung involvement and positive blood culture, or development of complications such as empyema requiring drainage and admission to the ICU [5-8]. Important comorbidities were regular alcohol consumption, dysphagia, chronic renal failure, neoplastic disease, urinary catheterization, secondary urinary tract infection among others [8-11]. Important social factors included the assessment of the family caregiver’s involvement, active and early involvement of the family in the discharge process, effective communication with the family, the provision of adequate information and education during the discharge process among others [4]. Within the presented study, we were able to evaluate both, factors focusing on the acute illness on admission and during follow up and social factors. In multivariate models, we identified independent LOS predictors which allowed individual LOS prediction. Thereby, our study expands previous efforts and proposes a clinical prediction rule, which may help physician to better estimate LOS in patients.

Our study population had a mean LOS of 9.8 days, which is longer than in most similar cohorts in the United States, but similar to other European centers. Importantly, the mean age of the population was 72 years of age with a high burden of comorbidities. As many patients needed post-acute care nursing assistance, LOS was not only dependent on the resolution of the acute disease, but also on organizational reasons. In line with this, we found that being a nursing home resident or needing regular nursing assistance at home was an independent predictor for LOS. Also, younger patients may present with a much more pronounced inflammatory reaction, but need shorter LOS compared to older patients. This is also in accordance with previous studies that found no difference in LOS despite reducing antibiotic courses with the use of a procalcitonin algorithm [12,14-16].

Increasing health care cost put an important burden on all health care systems. Inpatient management is up to 20 times more expensive than outpatient treatment [11,17-20]. Safely reducing the number of inpatient days is cost-effective and important from a societal perspective. As a result of continuous research organizational and financial pressure [21-29], LOS in CAP patients has continuously been declining in the past 20 years while maintaining and improving quality of care [20,30]. Importantly, previous research suggested that early discharge planning is effective and has the potential to markedly reduce LOS [31,32]. Our predictive rules may help clinicians to optimize discharge planning by indicating the expected LOS and indentifying important factors influencing LOS.

An interesting finding of our study was that private insurance status was not associated with LOS. This was also true when adjusting for age, complications and other potential confounders. This suggests that although Switzerland still had a pay-for-service health care system, patients with non-private insurance received similar treatment in regard to LOS compared to privately insured patients and there was no evidence of “extended-hospitalization” of privately insured patients.

Our study has several limitations. First, as a secondary analysis, we did not evaluate all known predictive factors for LOS such as respiratory acidosis, dysphagia, urinary catheterisation and secondary urinary tract infection and several social factors. Similarly, time to antibiotics and corticosteroid use was not assessed which may influence LOS. Second, this study only included Swiss hospitals which may limit external validity for other countries. Also, we did not assess time to clinical stability where patients could have been discharged if post-acute care facilities were available without limitations. Thus, our prediction rule may not apply unconditionally to other health care systems. LOS in this study was shorter than the average LOS for patients with CAP in Switzerland in the same years, but still higher than reported in similar CAP cohorts within the United States [3]. Third, our result may not be valid in all patients with CAP as the original ProHOSP trial had exclusion criteria, such as immune-suppression and dementia. Thus, we consider this study more hypothesis-generating than definite and future studies must validate our findings. Importantly, we encourage future interventional studies to investigate whether the use of our score for LOS prediction translates into shorter LOS without adverse effects on patient’s outcomes.

## Conclusion

In conclusion, we created a baseline and a follow-up prediction rule that accurately estimated LOS in CAP patients. If confirmed in future trials, knowledge of these factors may help to improve discharge management and avoidance of prolonged hospitalizations due to non-medical reasons.

## Competing interest

No commercial sponsor had any involvement in design and conduct of this study, namely collection, management, analysis, and interpretation of the data; and preparation, decision to submit, review, or approval of the manuscript.

MCC, WA, BM and PS received support from BRAHMS/Thermofisher to attend meetings and fulfilled speaking engagements. BM has served as a consultant and received research support. All other authors declare that they have no competing interests.

## Authors’ contributions

PS, MCC, WZ and BM had the idea, wrote the protocol and initiated the study. ISW, WZ, BM and PS managed the trial and collected data. ISW and PS performed the statistical analyses. ISW and PS drafted the manuscript and all authors amended and commented on the manuscript. All authors approved the final version.

## Funding sources

The initial trial was supported by grant SNF 3200BO-116177/1 and by grant SNF 32003B_135222 from the Swiss National Science Foundation. Dr. Schuetz was supported by a research grant from the Swiss Foundation for Grants in Biology and Medicine (Schweizerische Stiftung für medizinisch-biologische Stipendien, SSMBS, PASMP3-127684/1). Dr. Christ-Crain was supported by a grant of the Swiss National Science Foundation (PP00P3-12346).

## Pre-publication history

The pre-publication history for this paper can be accessed here:

http://www.biomedcentral.com/1471-2466/12/21/prepub
